# Planar silver nanowire, carbon nanotube and PEDOT:PSS nanocomposite transparent electrodes

**DOI:** 10.1088/1468-6996/16/2/025002

**Published:** 2015-03-23

**Authors:** Andrew J Stapleton, Soniya D Yambem, Ashley H Johns, Rakesh A Afre, Amanda V Ellis, Joe G Shapter, Gunther G Andersson, Jamie S Quinton, Paul L Burn, Paul Meredith, David A Lewis

**Affiliations:** 1 Flinders Centre for NanoScale Science and Technology, Flinders University, GPO Box 2100, Adelaide, SA, Australia; 2 Centre for Organic Photonics and Electronics, The University of Queensland, Brisbane, Queensland 4072, Australia

**Keywords:** transparent electrode, organic solar cell, silver nanowire, carbon nanotube, nanocomposite

## Abstract

Highly conductive, transparent and flexible planar electrodes were fabricated using interwoven silver nanowires and single-walled carbon nanotubes (AgNW:SWCNT) in a PEDOT:PSS matrix via an epoxy transfer method from a silicon template. The planar electrodes achieved a sheet resistance of 6.6 ± 0.0 *Ω*/□ and an average transmission of 86% between 400 and 800 nm. A high figure of merit of 367 *Ω*^−1^ is reported for the electrodes, which is much higher than that measured for indium tin oxide and reported for other AgNW composites. The AgNW:SWCNT:PEDOT:PSS electrode was used to fabricate low temperature (annealing free) devices demonstrating their potential to function with a range of organic semiconducting polymer:fullerene bulk heterojunction blend systems.

## 1. Introduction

Optoelectronic devices such as flat panel displays [1, 2], solar cells and light emitting diodes [3–5] generally rely on transparent electrodes such as indium tin oxide (ITO) for optical transparency and conductivity. ITO has become the most utilized material for transparent electrodes due to its acceptable sheet resistance (15–60 *Ω*/□) and high optical transparency (>90%) [6]. However, the life-time-cost-analysis of ITO shows that up to 87% of the energy required to fabricate conventional organic photovoltaic (OPV) devices is attributable to ITO fabrication [7, 8]. In addition to cost, the high processing temperatures (up to 400 °C) required to achieve optimal conductivity and its brittleness, are major impediments for applications in flexible applications and reel-to-reel processing, hence there has been considerable research into alternative transparent electrode materials.

Typically, with alternative transparent conducting electrodes there is a trade-off between optical transparency and conductivity and hence a ‘figure of merit’ is used to compare transparent electrodes. This figure of merit utilizes the sheet resistance of the electrode as well as the optical transmission at 550 nm, a larger value is an indication of better performance as a transparent conductor [9]. The figure of merit is derived from rearrangement of equation (1) and shown in equation (2), where *T* is the percent transmission at the wavelength of 500 nm and *R*_sh_ is the sheet resistance in *Ω*/□.1
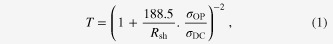
or2
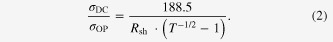



The value of the figure of merit for ITO on glass is typically between 30 and 320 *Ω*^−1^, depending on processing conditions, and is approximately 290 *Ω*^−1^ for commercial supplies [10]. However, the figure of merit of ITO on flexible substrates is around 40 *Ω*^−1^ due to a lower conductivity as a result of the lower annealing temperatures.

There have been reports of a number of different approaches for the fabrication of ITO-free transparent electrodes, including conducting polymer layers [11], ultrathin metal films [12], doped metal oxides (such as aluminium-doped zinc oxide) [13], metal oxide–silver–metal oxide (MAM) stacks [14], and silver nanowire (AgNW) based electrodes [15]. AgNW electrodes are a particularly attractive approach to transparent conducting electrodes due to the ease of nanowire manufacture, scalable solution processability [16–19], high conductivity and mechanical ductility [20, 21]. However, the relatively large diameter of the nanowires can result in high surface roughness depending on the processing conditions. For example, the authors have recently reported an interwoven silver nanowires and single-walled carbon nanotubes (AgNW:SWCNT) network with a sheet resistance in the range 4–20 *Ω*/□ and up to 82% *T* specular transparency [22]. However, the electrodes were not suitable for applications in thin flexible electronics due to the surface roughness of the interwoven nanowire networks.

A number of approaches to the fabrication of planar nanocomposite electrodes have been reported recently [18, 23–25]. The figure of merit was calculated from reported sheet resistances and optical transmissions of planar/smooth AgNW networks and found to be in the range of 100–240 *Ω*^−1^ [23–26]. Planarizing the surface onto which the active layer is to be deposited requires the network to be embedded into a planar polymer matrix, and this can result in non-conductive areas of up to 10 *μ*m between the AgNW network at the electrode surface. Gaynor *et al* [23] embedded AgNWs into a poly(3,4-ethylenedioxythiophene):poly(styrenesulfonate) (PEDOT:PSS) layer with a stamping technique to achieve smooth transparent electrodes. Yu *et al* [21] have also produced planar AgNW/polyacrylate electrodes with a surface roughness of less than 5 nm via a lift-off procedure from a glass substrate. Later work by the same group produced stretchable AgNW/polyacrylate composites with a sheet resistance of 7.4 *Ω*/□ a and a transmittance of around 80% by using the same lift-off procedure [25]. Akter and Kim [24] also used a lift-off technique to create stretchable planar transparent electrodes of AgNWs embedded in polydimethylsiloxane (PDMS) with >80% transmittance and an average sheet resistance of >35 *Ω*/□.

To achieve a high visible light transmission of AgNW transparent conducting electrodes it is necessary to minimize the amount of interwoven nanowires in the film. However, poor connectivity and low concentration increases the sheet resistance and reduces the extraction efficiency of free charges generated within the active layer, and hence there is a trade-off between these two factors [27]. Incorporation of a second less absorbing/reflective interpenetrating conducting material should be beneficial to bridge the AgNW component. Despite the encouraging transparency and sheet resistance properties of the first AgNW:SWCNT transparent electrodes, an important development requirement is to be able modify the electrode processing conditions to enable a planarized surface of the electrode for device fabrication.

Herein we report the production of a high figure of merit planarized (<5 nm rms roughness) AgNW:SWCNT transparent electrode embedded in a thin PEDOT:PSS using an epoxy adhesion lift-off technique. We demonstrate its use in OPV devices with a direct comparison to standard ITO-based solar cells.

## 2. Materials and methods

AgNWs were purchased from Seashell Technologies (San Diego, USA), which were supplied as a suspension (20.4 mg mL^−1^) in 2-propanol (IPA). An aliquot of the AgNW suspension was diluted to 0.1 mg mL^−1^ with IPA and stored until use. Carboxylate functionalized (P3 type) SWCNTs with purity of >90% were purchased from Carbon Solutions (California, USA). The carboxylate functionalized SWCNT (50 mg) were treated in 3 M HNO_3_ heated at reflux for 12 h before being collected using a filter (0.45 *μ*m polycarbonate, Millipore). It has been shown that mild acid treatment of the SWCNTs improves aqueous dispersibility and performance of interwoven AgNW:SWCNT films [22, 28]. A sample of the treated SWCNTs was suspended in water via probe sonication (Sonics Vibracell^™^) at 40% amplitude for 2 min before being diluted to a concentration of 0.25 mg mL^−1^ with deionized water. The morphology of the AgNWs and SWCNTs used in this work have been reported previously [22]. The lengths of the as-purchased AgNWs were shown to be in the order of 5–50 *μ*m with a diameter of approximately 100–200 nm, while after the mild oxidation treatment the SWCNTs were found to exist in bundles with a bundle diameter of 5–15 nm.

Interwoven networks were prepared via vacuum filtration through mixed cellulose ester membranes (MF-Millipore Membrane, USA, mixed cellulose esters, hydrophilic, 0.45 *μ*m, 47 mm). Various volumes of pre-prepared AgNW (0.1 mg mL^−1^) and SWCNT (0.25 mg mL^−1^) solutions were added to 300 mL of deionized water so that approximately a surface loading of 125 mg m^−2^ of AgNWs was achieved in the final nanocomposite electrode. Patterned electrodes were formed by placing a smaller pore size mixed cellulose ester template (MF-Millipore Membrane, mixed cellulose esters, hydrophilic, 0.025 *μ*m, 47 mm) under the 0.45 *μ*m membrane during filtration (figure 2(a)). The 0.025 *μ*m patterning template had the required electrode pattern cut from the middle of the membrane. After filtration the patterned electrodes were then placed on pre-cleaned silicon substrates (sonicated in acetone and then 2-propanol for 15 min each). The silicon and patterned electrodes were then heated at 80 °C under 0.16 kg cm^−2^ of pressure in a vacuum oven (Memmert, Germany) for 30 min (figure 2(b)). The mixed cellulose ester filter paper was subsequently removed by dissolution in acetone for 60 min leaving behind the patterned AgNW:SWCNT nanocomposite on the silicon substrate (figure 2(c)). Subsequently, a PEDOT:PSS solution (Clevios^™^, approximately 1% aqueous suspension) mixed with 33 v/v% 2-propanol and 10 v/v% sorbitol (200 *μ*L) was spin-coated on top of the AgNW:SWCNT nanocomposite at 500 rpm for 5 s then at 3000 rpm for 30 s. The nanocomposite was then annealed at 140 °C for 10 min (figure 2(d)). Epotek 301 epoxy resin (50 *μ*L, *T* = 99%) was then placed on top of the PEDOT:PSS coated AgNW:SWCNT electrode. A glass substrate for transfer was placed on top of the epoxy to create an AgNW:SWCNT:PEDOT:PSS/epoxy/glass stack. The stack was then heated at 65 °C for 1 h in an oven (Memmert, Germany) to cure the epoxy. The stack was then put into liquid nitrogen in order to cleave the silicon/PEDOT:PSS interface, which occurs due to thermal stress, and this process results in a planar conducting surface on the glass substrate.

**Figure 1.**
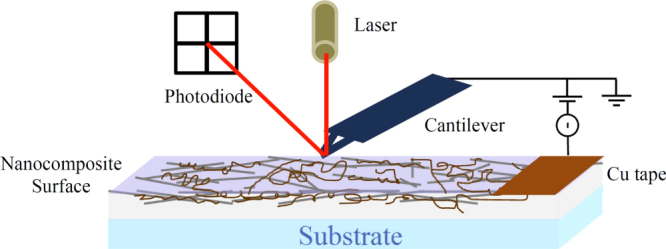
PF-TUNA experimental setup.

Sheet resistance measurements were performed using a four point probe (KeithLink^®^ Technology, New Taipei City, Taiwan). The values reported were an average of 10 measurements on two separate 64 mm^2^ samples. Transmission and reflectivity were measured on samples (25 mm^2^) using a Perkin–Elmer LAMBDA 950 UV/vis/NIR spectrophotometer with integrating sphere. The average transmission reported was for a wavelength range between 400 and 800 nm. Scanning electron microscopy (SEM) images were acquired using a CamScan MX2500 microscope (CamScan Optics, Cambridge, UK) working at an accelerating voltage of 10 kV and a distance of 10 mm. Topographical atomic force microscopy (AFM) images were acquired using a Bruker Multimode AFM with Nanoscope V controller. NSC15 Mikromasch silicon tapping mode probes with a nominal spring constant of 40 N m^−1^, resonant frequency of 325 kHz and tip diameter equal to 20 nm were used. AFM images were acquired in tapping mode with all parameters including set-point, scan rate and feedback gains adjusted to optimize image quality and minimize imaging force. Root mean square roughness (R_rms_) values were obtained from plane-fitted image scans of 10 *μ*m^2^. The electrical conductivity of the planar AgNW:SWCNT electrodes were mapped using peak force tunnelling AFM (PF-TUNA) [29] on a Bruker Multimode AFM with Nanoscope V controller. The software used to acquire all AFM data was control software version 8.15. The cantilevers used to obtain the PF-TUNA images were Bruker SCM-PIT conducting probes with a spring constant of 1–5 N m^−1^. The entire cantilever and tip was coated with 20 nm of platinum and iridium resulting in a total tip diameter of approximately 40 nm. The sample surface was electrically connected via copper tape and the instrumental setup shown in figure 1. PF-TUNA imaging parameters including set-point, scan rate, feedback gains, current sensitivity and applied bias were adjusted to optimize height and current image quality. The scanner was calibrated in *x*, *y* and *z* directions using silicon calibration grids (Bruker model numbers PG: 1 *μ*m pitch, 110 nm depth and VGRP: 10 *μ*m pitch, 180 nm depth).

For testing the AgNW:SWCNT:PEDOT:PSS electrodes in devices, two types of cells were fabricated using two different organic semiconductor blends. The devices had the following structures: (1) glass/AgNW:SWCNT/MoO_*x*_/poly(3-*n*-hexylthiophene-2, 5-diyl) (P3HT):phenyl-C_61_-butyric acid methyl ester (PC_60_BM)/Al, and (2) glass/AgNW:SWCNT/MoO_*x*_/poly(*N*-9″-heptadecanyl-2, 7-carbazole-*alt*-5, 5-(4′,7′-di-2-thienyl-2′,1′,3′-benzothiadiazole)) (PCDTBT):(6, 6)-phenyl C_70_-butyric acid methyl ester (PC_70_BM)/Al. The device structures are inset into figure 6. For comparison, devices of the same structure were also fabricated on commercial ITO.

Pre-patterned ITO substrates (Xinyan Technology) were cleaned using Alconox (Alconox) in deionized water. The substrates were then rinsed several times with deionized water and ultra-sonicated in the same for 5 min. It was followed by consecutive ultra-sonication in acetone and isopropanol for 10 min each. The substrates were dried using a nitrogen gun. After this, all steps of device fabrications and testing for all types of devices were carried out in a nitrogen environment (MBraun glove box, O_2_ < 0.1 ppm; H_2_O < 0.1 ppm). A thin film of MoO_*x*_ (Sigma Aldrich) was deposited on the planarized AgNW:SWCNT film using thermal evaporation at a pressure ∼1 × 10^−6^ mbar. For the P3HT:PCBM photoactive layer, a blend of P3HT:PCBM (1:1 w/w) was prepared by mixing equal amounts of individual solutions of P3HT (Merck, Mw = 94 kDa, polydispersity index (PDI) = 1.9) and PC_60_BM (American Dye Source) in 1, 2-dichlorobenzene (DCB) (anhydrous grade). Both individual solutions had a concentration of 30 mg mL^−1^. The P3HT:PC_60_BM blend was then filtered (0.22 *μ*m PTFE filter, Membrane Solutions) and spin-coated (500 rpm for 3 s, then 1400 rpm for 17 s) on top of the MoO_*x*_ layer. The PCDTBT:PC_70_BM active layer was prepared by mixing a 6 mg mL^−1^ solution of PCDTBT (SPJC, Canada, Mw = 122 kDa, PDI = 5.4) in DCB in a 24 mg mL^−1^ solution of PC_70_BM (Nano-C) in DCB. The PCDTBT:PC_70_BM (1:4 w/w) blend was then spin-coated (500 rpm for 3 s, then 800 rpm for 77 s) onto the MoO_*x*_ layer. Both the P3HT:PCBM (1:1 w/w) and the PCDTBT:PC_70_BM (1:4 w/w) films were dried at 60 °C for 20 min on a hot plate. Finally, a thick layer of Al was deposited by thermal evaporation at a pressure of ∼1 × 10^−6^ mbar to complete the fabrication. The final devices had an active area of 0.2 cm^2^, which was defined using a shadow mask when evaporating the cathode.

Finally, device characterization was undertaken using an Abet Triple-A (Abet Technologies) solar simulator. The solar mismatch of the Xenon lamp (550 W Oriel) spectrum was minimized using an AM1.5G filter. Light intensity at ∼100 mW cm^−2^ AM1.5G was calibrated with a National Renewable Energy Laboratory (NREL)-certified standard silicon photodiode (2 cm^2^), with a KG5 filter. A Keithley^®^ 2400 source measurement unit in a 4-wire configuration setup was used for current density-voltage measurements.

## 3. Results and discussion

### 3.1. Preparation of planar AgNW:SWCNT:PEDOT:PSS electrodes

**Figure 2.**
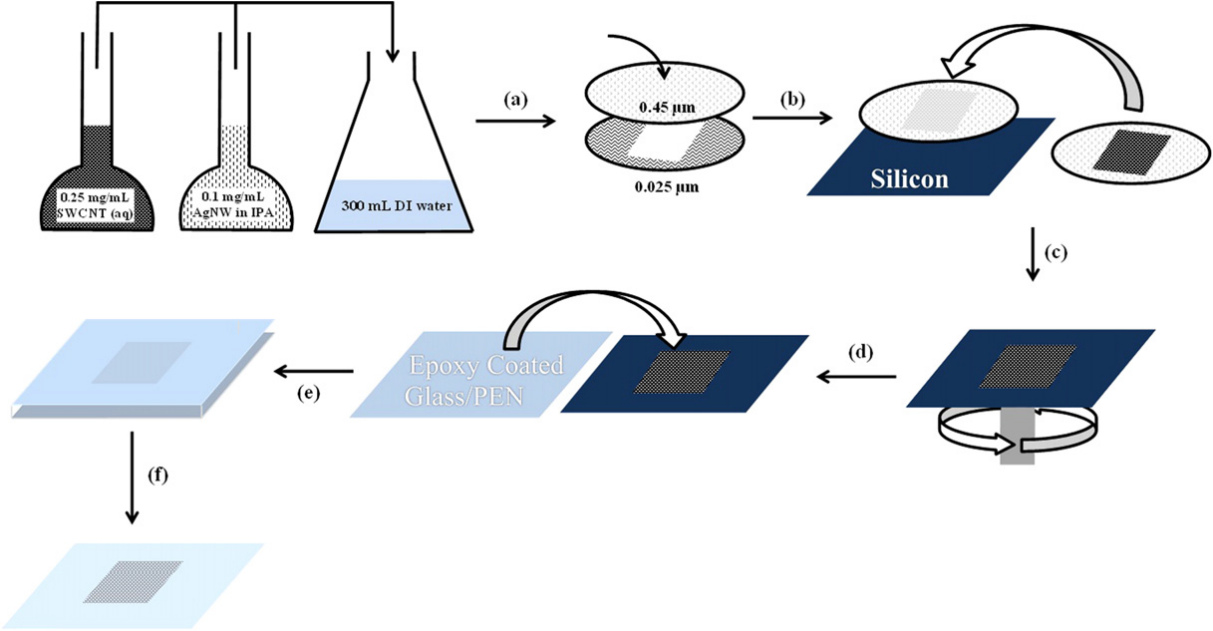
Schematic outlining the fabrication of planar AgNW:SWCNT:PEDOT:PSS nanocomposite electrodes on a glass substrate. (a) Substrate patterning through cellulose ester membrane, (b) transfer to silicon substrate, (c) spin-coating of PEDOT:PSS solution, (d) creation of epoxy sandwich structure, (e) curing the epoxy, (f) liquid nitrogen separation of electrode from silicon template. DI stands for deionized and PEN represents polyethylene naphthalate.

AgNW:SWCNT:PEDOT:PSS planar electrodes (20 wt% SWCNT) were fabricated via the process steps outlined in figure 2 to achieve a surface loading of AgNW of 125 mg m^−2^ with 20 wt% SWCNT. A weight fraction of 20 wt% SWCNTs was chosen as it has been shown previously to retain low sheet resistivity without impacting optical transparency in AgNW and SWCNT interwoven networks [22]. The process comprised the following steps: (i) making a mixed dispersion of AgNW and SWCNT in deionized water; (ii) filtration through a masked cellulose ester membrane to create the desired interwoven pattern; (iii) pressing the interwoven pattern onto a silicon substrate and dissolving the membrane; (iv) coating the silicon and the interwoven electrode with PEDOT:PSS and drying; and (v) addition of an epoxy base layer to adhere to a glass substrate. Given the ‘open’ nature of the AgNW:SWCNT mat it was necessary to fill in the gaps to prevent the epoxy layer adhering to the underlying Si wafer. PEDOT:PSS was chosen as the in-filling/planarization component layer due to its conductivity [30]. It was also expected that the PEDOT:PSS layer would act as an intermediary charge collection pathway, with charges migrating towards, and being collected by, the interwoven AgNW:SWCNT network. 10 v/v% sorbitol was mixed with the PEDOT:PSS as it has been shown to improve the conductivity and also modify the work function [31]. It was found that this fraction of sorbitol was sufficient to align the work function of the electrode with the active layers of the OPV device and avoid s-shaped *J*–*V* curves.

### 3.2. Electrode properties

**Figure 3.**
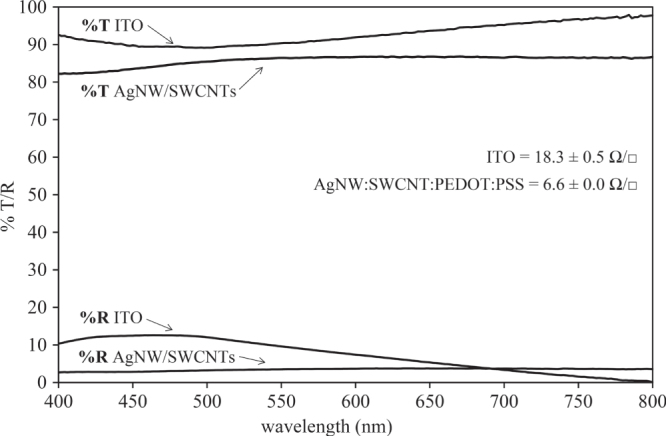
Transmission (%*T*) and reflectivity (%*R*) of an ITO and planar AgNW:SWCNT:PEDOT:PSS nanocomposite electrode corrected for the substrate contribution. The sheet resistances, shown on the right, are an average of 15 measurements on 3 separate 25 mm^2^ samples.

The transmission of the resulting electrode was relatively flat across the wavelength range 400–800 nm with an average transmission of 86.0 ± 1.4% and an average reflectivity of 3.4 ± 0.3% as shown in figure 3. In contrast, the commercial ITO electrode had an average transmission of 93.0 ± 7% and average reflectivity of 7 ± 4%. Typically the planarizing/adhesion layer used to create the smooth electrode surface improves the overall transmission of the electrode and thus the figure of merit substantially.

The measured average sheet resistance of the AgNW:SWCNT:PEDOT:PSS electrode (6.5 *Ω*/□) was found to be almost half of that reported by Gaynor *et al* [23] for AgNW-only electrodes (12 *Ω*/□). Importantly, our AgNW:SWCNT electrodes had an average sheet resistance much lower than commercial ITO electrodes (18.3 *Ω*/□).

The figure of merit (from equation (2)) for the AgNW:SWCNT:PEDOT:PSS electrodes was determined to be 367 *Ω*^−1^, (table 1), which is only matched by the non-planar AgNW-only electrodes [9, 32]. The properties of the AgNW:SWCNT:PEDOT:PSS and ITO electrode are summarized in table 1.

**Table 1.** Summary of properties of ITO and AgNW:SWCNT:PEDOT:PSS electrodes.

	Average %*T* (400–800 nm)	Average %*R* (400–800 nm)	Sheet resistance (*Ω*/□)	Figure of merit (*Ω*^−1^)
AgNW:SWCNT:PEDOT:PSS	86.0 ± 1.0	3.4 ± 0.3	6.6 ± 0.5	367
ITO	93.0 ± 7.3	7.2 ± 4.3	18.3 ± 0.5	292

**Figure 4.**
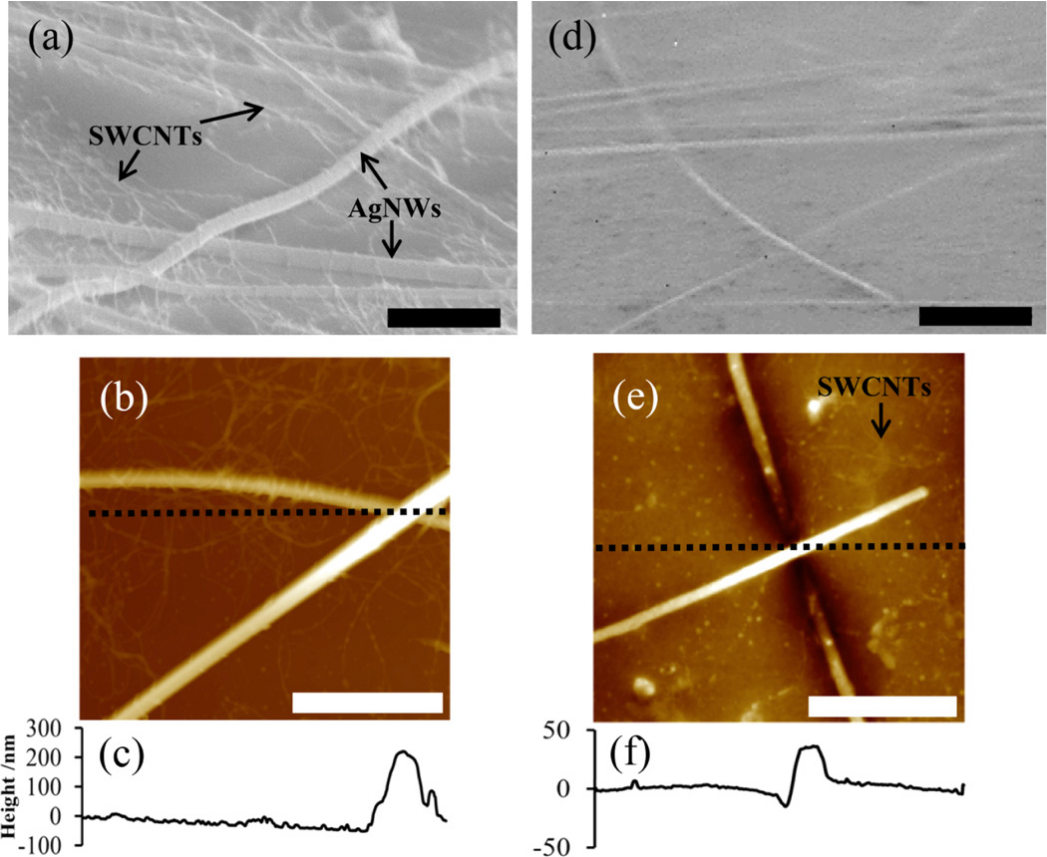
SEM images of (a) non-planarized AgNW and SWCNTs on a glass substrate and (d) the AgNWs:SWCNT:PEDOT:PSS electrode after the planarization. AFM of (b) non-planarized AgNW and SWCNTs on glass and (e) AgNWs and SWCNTs after the planarization process. The height profiles along the dotted lines in (b) and (e) are shown in (c) and (f), respectively. All scale bars are 2 *μ*m.

SEM (figures 4(a) and (d)) and AFM (figures 4(b) and (e)) images reveal that before planarization (figure 4(a)), the AgNW:SWCNT electrodes have a complex surface topography and exists as interwoven networks of AgNWs and SWCNTs. The reason for the close association between the AgNWs and the SWCNTs has been observed previously and has been determined to be due to a solution phase, interaction between the AgNWs and the side walls of the SWCNTs prior to deposition onto the cellulose ester membranes [22]. After completion of the planarization process however, the SEM reveals a significantly smoother surface and all of the AgNWs and SWCNTs are embedded into the PEDOT:PSS and epoxy supporting layer (figure 4(d)). The change in surface morphology upon planarization was also monitored via tapping mode AFM and the images are shown in figure 4(b) for the unplanarized and (e) for the planarized electrodes. Figures 4(c) and (f) are the height profiles along the dotted lines in the AFM images of figures 4(b) and (e). Figure 4(e) shows the surface morphology of the planarized AgNW:SWCNT:PEDOT:PSS electrode surface; the height profile is significantly smoother than for a non-planarized AgNW:SWCNT electrode (figure 4(c)) despite the fact that the height profile is positioned over the crossing point of two AgNWs. The roughness (Rq) of the planarized AgNW:SWCNT:PEDOT:PSS electrode over a plane-fitted image scan of 10 *μ*m^2^ was measured to be 3.5 nm. Interestingly, we can still observe what are presumably SWCNTs in the top right hand quadrant of figure 4(d) (indicated by the arrow). Since the SWCNTs were observed to be present at the top interface we believe they also participate in charge collection, passing extracted charges to the more conductive AgNWs.

**Figure 5.**
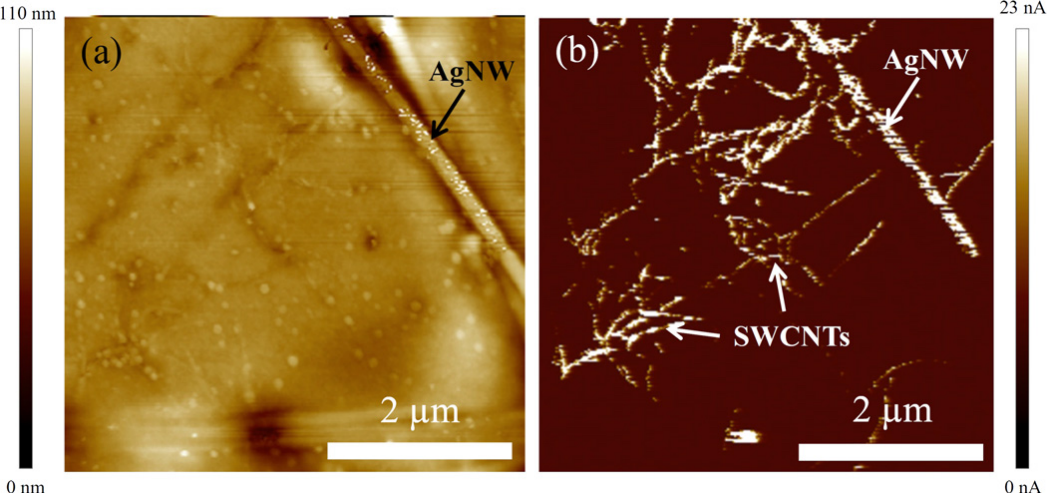
(a) Height and (b) peak force current map of planarized AgNW:SWCNT electrode surface with a bias voltage of 2 V.

PF-TUNA provides evidence of the ability for SWCNTs to contribute to the charge collecting ability of the nanocomposite electrode as a secondary charge-collecting network. Figure 5(a) shows the height image of the planarized electrode surface where a silver nanowire is observed crossing the top right hand quadrant of the image. Figure 5(b) shows the peak force current map of the planarized electrode surface at a 2 V applied bias. It is apparent from figure 5(b) that the SWCNTs are electrically connected to the AgNW and are present in a significant density at the surface of the electrode. Importantly, the SWCNTs and AgNWs are not covered by any significant amount of the epoxy that is used as an adhesive layer to adhere the AgNW:SWCNT network to a glass substrate. When PEDOT:PSS was not used to fill the gaps in the AgNW:SWCNT network the electrode became resistive and no AgNWs were able to be identified in the current map of the surface.

### 3.3. Device fabrication and properties

**Figure 6.**
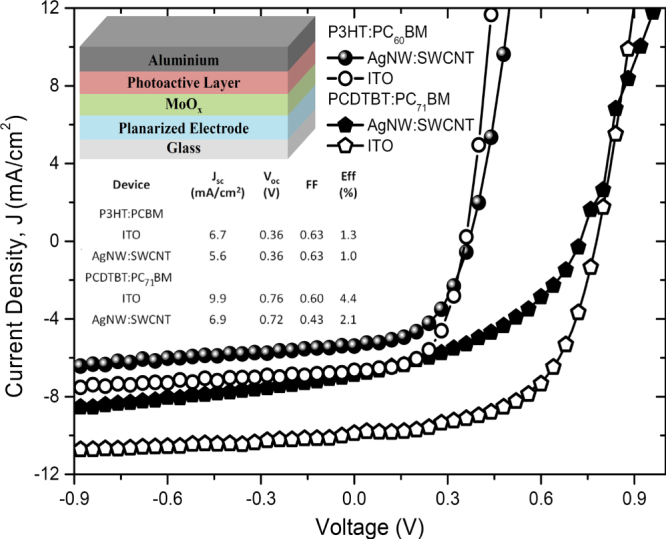
*J*–*V* characteristics of best OPV devices on planarized AgNW:SWCNT:PEDOT:PSS nanocomposite transparent electrodes and ITO with P3HT:PC_60_BM and PCDTBT:PC_70_BM active layers and performance parameters. Inset shows structure of an OPV device.

OPV devices were successfully fabricated on the planarized AgNW:SWCNT:PEDOT:PSS electrodes using P3HT:PC_60_BM and PCDTBT:PC_71_BM active layers. Current density (*J*) versus voltage (*V*) characteristics of the best devices including their parameters are shown in figure 6. Inset of figure 6 shows the simple device structure chosen for this work. It has been shown that this structure works well with ITO electrodes [33]. The P3HT:PC_60_BM devices reached an efficiency of 1.0% while PCDTBT:PC_70_BM devices reached an efficiency of 2.1%. Some parameters of the device on AgNW:SWCNT:PEDOT:PSS electrodes are comparable to those on ITO showing the potential of AgNW:SWCNT:PEDOT:PSS as an alternative to ITO. Average device parameters including the open circuit voltage (*V*_OC_), short circuit current density (*J*_SC_), fill factor (FF) and efficiency are given in the supplementary information. Averages for the parameters were taken over at least five devices in each type and standard deviations are included as errors. It should be noted that the results presented for devices performance are preliminary results. A significant improvement in efficiency is expected by optimizing processing conditions and optimizing thicknesses of the constituent layers.

## 4. Conclusions

Planar nanocomposite electrodes based on interwoven AgNWs, SWCNTs and PEDOT:PSS have been fabricated with a superior figure of merit (367 *Ω*^−1^)—a combination of transparency and conductivity—than ITO (267 *Ω*^−1^) on glass. The surface of the electrode has an average surface roughness of <5 nm enabling the fabrication of OPV devices. The PEDOT:PSS was found to be an important inclusion to the structure and without it the electrode surface became resistive, which has been ascribed to epoxy covering the entire surface. PF-TUNA showed that the SWCNTs were active participants in charge collection and distribution within the electrode with clear evidence of intimate contact between the SWCNT and the AgNWs. The AgNW:SWCNT:PEDOT:PSS electrodes were used to fabricate low temperature (non-annealed) devices using two different bulk heterojunction layers resulting in an average efficiency of 0.9 ± 0.1% (75% the efficiency of the equivalent ITO device) and 1.8 ± 0.2% (50% the efficiency of the equivalent ITO device) for OPV devices fabricated with P3HT:PC_60_BM and PCDTBT:PC_70_BM, respectively. Therefore demonstrating the potential for the AgNW:SWCNT:PEDOT:PSS electrode to function with a range of organic semiconducting polymer:fullerene bulk heterojunction blend systems.
